# Global Mental Health Meets Social Innovation: the HOW matters

**DOI:** 10.1192/j.eurpsy.2023.712

**Published:** 2023-07-19

**Authors:** R. P. F. Wolthusen, P. Andrä

**Affiliations:** ^1^Psychiatry, Duke University Medical Center, Durham, United States; ^2^University Children Hospital, Zurich, Switzerland

## Abstract

**Introduction:**

Mental health conditions are rising globally, and COVID-19 has exacerbated the situation. We often think that massive money investments and training of specialized mental health providers, such as psychiatrists, will help alleviate the demand-supply challenge. But the reality is different. Despite all efforts over the last years, the mental health treatment gap, the percentage difference between the number of people needing treatment for mental illness and the number of people receiving treatment, is still 50+% in countries like Germany. The investment of money and the training of specialized mental health providers alone will not be sudcient to decrease this number.

**Objectives:**

We need to learn from and with partners from low- and middle-income countries (LMIC), in which a shortage of resources has prevented a signihcant investment in mental health but also has inspired the innovation and implementation of novel approaches to decrease the mental health treatment gap. This reshaped approach allows us to move from Northern Ventriloquism (high-income countries teach LMIC what to do) to honest cross-cultural bidirectional learning. Furthermore, it will hll the “how” gap.

**Methods:**

We know WHY we should act in the (global) mental health field. We also know WHAT we should do. The main question remaining is HOW we can implement any of the activities. To fill this “how” gap, the Dresden-based NGO On The Move e.V. designed an annual 8-week program funded by the European Union, which centers around a global mental health and social innovation curriculum and aims to create spaces of empowerment towards mentally healthier communities. Our participants come from four higher education institutions in Germany, Ghana, and Kenya.

**Results:**

The program, which was recently awarded the TU Dresden internationalization award in the category “Innovative International Research Cooperation,” encourages participants to learn from and with each other. To enable an holistic approach to mental health and diversify the pool of mental health champions, the program includes participants from all fields. Since the start of the program, hundreds of culturally sensitive mental health-related Youtube videos have been recorded and distributed widely in the communities of the participants. The number of participant-led advocacy events has also increased.

**Conclusions:**

Contextually, we will discuss core concepts, such as human-centered and community-based approaches, and how they relate to filling the “how” gap in our presentation. We might not have a blueprint of solutions in terms of decreasing the mental health treatment gap; however, our recommendations can support innovative and customized solutions. From a process perspective, we will compare existing global mental health training curricula with our curriculum and highlight transcultural learning opportunities; we will also discuss the elements of our program that empower our trainees.

**Disclosure of Interest:**

None Declared

